# The relationship of depression in asthma–chronic obstructive pulmonary disease overlap syndrome

**DOI:** 10.1371/journal.pone.0188017

**Published:** 2017-12-12

**Authors:** Jun-Jun Yeh, Cheng-Li Lin, Wu-Huei Hsu, Chia-Hung Kao

**Affiliations:** 1 Ditmanson Medical Foundation Chia-Yi Christian Hospital, Chiayi, Taiwan; 2 Chia Nan University of Pharmacy and Science, Tainan, Taiwan; 3 Meiho University, Pingtung, Taiwan; 4 Management Office for Health Data, China Medical University Hospital, Taichung, Taiwan; 5 College of Medicine, China Medical University, Taichung, Taiwan; 6 Graduate Institute of Clinical Medical Science and School of Medicine, College of Medicine, China Medical University, Taichung, Taiwan; 7 Division of Pulmonary and Critical Care Medicine, Department of Internal Medicine, China Medical University Hospital, Taichung, Taiwan; 8 Department of Nuclear Medicine and PET Center, China Medical University Hospital, Taichung, Taiwan; 9 Department of Bioinformatics and Medical Engineering, Asia University, Taichung, Taiwan; Telethon Institute for Child Health Research, AUSTRALIA

## Abstract

**Purpose:**

To clarify the relationship between asthma–chronic obstructive pulmonary disease overlap syndrome (ACOS) and depression.

**Methods:**

We identified 10,911 patients who received an ACOS diagnosis and concurrent treatment between January 2000 and December 2009. Subjects without ACOS were included in the non-ACOS cohort (*n* = 10,911). Cox proportional hazard regression analysis was performed to compare the risk of depression between the ACOS and non-ACOS cohorts.

**Results:**

The risk of depression was higher in the ACOS cohort than in the non-ACOS cohort (adjusted hazard ratios (aHRs) = 1.67, 95% confidence interval [CI] = 1.48–1.88). In the ACOS cohort, the aHRs for depression were [2.44 (95% CI = 1.45–4.11); 2.36 (95% CI = 1.58–3.52)] in patients [aged 20–39 years; without comorbidity]. In the ACOS cohort, the aHRs for depression were 1.70 (95% CI = 1.51–1.93) and 1.84 (95% CI = 1.55–2.19) in patients without inhaled corticosteroids (ICSs) and oral steroids (OSs) use, respectively. Moreover, the aHRs for the risk of depression were 1.16 (95% CI = 0.95–1.41) and 1.12 (95% CI = 0.96–1.29) in patients with ICSs and OSs use, respectively.

**Conclusion:**

The risk of depression is higher in ACOS patients, even in those without comorbidities or in young adults. The events of the depression were not significant difference in patients receiving the ICSs/OSs between the ACOS and the non-ACOS cohorts.

## Introduction

The burden of depression has recently increased worldwide [1]. A worsening prognosis, multiple comorbidities, and readmission contribute to depression in patients with chronic diseases such as chronic obstructive pulmonary disease (COPD) [2, 3]. In addition, asthma is associated with depression in elderly people [4]. Therefore, recent studies have extensively investigated the relationship of depression with asthma and COPD. Asthma–COPD syndrome (ACOS) is a disorder in which a patient presents with symptoms of both asthma and COPD [5]. Until now, few studies published in English have reported the association of depression with ACOS.

Steroids are used for managing ACOS [5]. The pathophysiology of the neuropsychiatric sequelae of steroid treatment remains unclear. Decreases in central and peripheral serotonin secretion [6] are linked to steroid administration [7]. Hence, steroid administration has been postulated to cause depression by reducing serotonin levels. In addition, hypogonadism increases the risk of depression in patients with readmission [8, 9]. Bonala et al demonstrated that ICSs use increased the risk of depression [10]. By contrast, Passalacqua et al [11] reported that depression disappeared after the initial use of ICSs. Moreover, Steroid Treatment as Regular Therapy (START) study corroborated this observation [12]. The START study demonstrated that patients treated with ICSs had more favorable asthma control and significantly less depression, with trends of improvement in anxiety and behavioral disturbances [13]. Recently, van Boven et al [14] compared an ACOS cohort with a pure COPD cohort and observed no significant difference in the risk of depression between these two cohorts. The relationship between steroid use and depression in asthma/COPD is to be debated.

Aging poses a challenge to healthcare systems because of the high prevalence and impact of multiple morbidities in older adults [10]. ACOS is associated with older age [15]. The major components of metabolic syndrome [10] (e.g., hypertension, hyperlipidemia, and diabetes), anxiety, sleep disorder [16], alcohol-related disease, and coronary artery disease [17] are associated with depression. Moreover, these diseases [18] occur as comorbidities [14] in ACOS patients. In the present study, we investigated the relationship of oral steroids (OSs) [19, 20] and inhaled corticosteroids (ICSs) [19, 21] use with depression in ACOS patients by using a nationally representative sample.

## Methods

### Data source

This study was conducted using the Longitudinal Health Insurance Database (LHID), which contains the claims data of one million insurants selected from the National Health Insurance (NHI) program of Taiwan during 1996–2000. The NHI program is a national health insurance program that covers more than 99% of the 23 million citizens of Taiwan. The LHID comprises health claims data, such as the registry of beneficiaries, disease registry files (recorded based on the International Classification of Diseases, Ninth Revision, Clinical Modification [ICD-9-CM]), drug prescriptions, and other medical services, and this database is updated annually. To ensure the privacy of the insurants, the database is released for research purposes after the data have been deidentified.

### Ethics statement

The NHIRD encrypts patient personal information to protect privacy and provides researchers with anonymous identification numbers associated with relevant claims information, including sex, date of birth, medical services received, and prescriptions. Therefore, patient consent is not required to access the NHIRD. This study was approved to fulfill the condition for exemption by the Institutional Review Board (IRB) of China Medical University (CMUH104-REC2-115-CR2). The IRB also specifically waived the consent requirement.

### Data availability statement

The dataset used in this study is held by the Taiwan Ministry of Health and Welfare (MOHW). The Ministry of Health and Welfare must approve our application to access this data. Any researcher interested in accessing this dataset can submit an application form to the Ministry of Health and Welfare requesting access. Please contact the staff of MOHW (Email: stcarolwu@mohw.gov.tw) for further assistance. Taiwan Ministry of Health and Welfare Address: No.488, Sec. 6, Zhongxiao E. Rd., Nangang Dist., Taipei City 115, Taiwan (R.O.C.). Phone: +886-2-8590-6848. All relevant data are within the paper.

### Study population

In this population-based cohort study, we investigated the risk of depression in ACOS patients. ACOS patients were defined as those with coexisting COPD and asthma. The ACOS cohort included patients older than 20 years who had COPD (ICD-9-CM codes 491, 492, and 496) and concurrent physician-diagnosed asthma (ICD-9-CM code 493) between January 2000 and December 2009 [14]. The comparison cohort included subjects without ACOS who were propensity score matched [22] to the ACOS cohort at a ratio of 1:1 by sex, age, and comorbidities for avoiding the selection bias. Individuals with a history of depression were excluded. The outcomes of interest of this study were the occurrence of a depression event (ICD-9-CM codes 296.2–296.3, 300.4, and 311). We followed up these study subjects until withdrawal from the NHI program, occurrence of a depression event, or December 31, 2011.

This study also considered the effect of comorbidities and drug use. The depression-associated comorbidities considered in this study were hypertension (ICD-9-CM codes 401–405), diabetes (ICD-9-CM code 250), hyperlipidemia (ICD-9-CM code 272), ischemic heart disease (IHD; ICD-9-CM codes 410–414), sleep disorder (ICD-9-CM codes 307.4 and 780.5), anxiety (ICD-9-CM code 300.00), alcohol-related illness (ICD-9-CM codes 291, 303, 305, 571.0, 571.1, 571.2, 571.3, 790.3, and V11.3), vitamin d deficiency (ICD-9-CM 268), cancer (ICD-9-CM codes 140–208), tuberculosis (ICD-9-CM codes 010–018), rheumatoid arthritis (ICD-9-CM code 714), gout (ICD-9-CM code 274), viral hepatitis (ICD-9-CM code 070), and herpes simplex (ICD-9-CM code 054). The drug types considered were ICSs and OSs [20]. Drug use was defined as receiving a drug during follow-up and exposure to the drug for ≥30 days [6].

### Statistical analyses

The distribution of characteristics between the ACOS cohort and non-ACOS cohort is expressed as the mean and standard deviation (SD) for age and frequency and percentage for sex and comorbidities. Differences between the ACOS cohort and non-ACOS cohort were determined using the *t* test for age and the chi-square test for sex and comorbidities. To estimate the rate of depression, the incidence density was calculated for each cohort by dividing the number of depression events by the total follow-up years (per 1,000 person-years). The cumulative incidence curves were plotted using the Kaplan–Meier method, and the difference between the curves was tested using the log-rank test. Univariable and multivariable Cox proportional hazard models were used to estimate the hazard ratios (HRs) and corresponding 95% confidence intervals (CIs) for comparing the risk of depression between the ACOS cohort and non-ACOS cohort. The significance level was set at p < 0.05. Statistical analyses were performed using SAS 9.4 (SAS Institute Inc., NC, USA), and the incidence curves were plotted using R software (R Foundation for Statistical Computing, Vienna, Austria).

## Results

This study included 10,911 ACOS patients and 10,911 subjects without ACOS. The mean age (approximately 65 years), sex ratio (57.6% were men) and comorbidities were similar between the two cohorts (Table 1). A significantly higher proportion of patients in the ACOS cohort exhibited medicines, such as ICSs, and OSs, compared with subjects in the non-ACOS cohort.

**Table 1:** Comparison of demographic risk factors between the ACOS and non-ACOS cohorts.

	ACOS				
	No(N = 10911)		Yes(N = 10911)		
Variable	n	%	n	%	Standard difference^§^
**Sex**					
Women	4539	41.6	4630	42.4	0.02
Men	6372	58.4	6281	57.6	0.02
**Age, year**					
20–39	602	5.52	705	6.46	0.04
40–64	3840	35.2	4041	37.0	0.04
≥65	6469	59.3	6165	56.5	0.06
Mean (SD)	66.3(13.9)		65.0(14.5)		0.09
**Comorbidity**					
Hypertension	7124	65.3	6694	61.4	0.08
Diabetes	3046	27.9	2903	26.6	0.03
Hyperlipidemia	3725	34.1	3548	32.5	0.03
IHD	4732	43.4	4496	41.2	0.04
Sleep disorder	3194	29.3	3141	28.8	0.01
Anxiety	1144	10.5	1185	10.9	0.01
Alcohol-related illness	255	2.34	274	2.51	0.01
Vitamin D deficiency	5	0.05	6	0.05	0.004
Cancer	1303	11.9	1253	11.5	0.01
Tubeculosis	1009	9.25	1117	10.2	0.03
Rheumatoid arthritis	59	0.54	57	0.52	0.003
Gout	3053	28.0	3006	27.6	0.01
Viral hepatitis	906	8.30	992	9.09	0.03
Herpes simplex	378	3.46	397	3.64	0.01
Medicine					
Inhaled corticosteroids (ICSs)	459	4.21	2836	26.0	0.64
Oral steroids (OSs)	5294	48.5	8485	77.8	0.64

ACOS, asthma–COPD overlap syndrome; COPD, chronic obstructive pulmonary disease; IHD, ischemic heart disease; chi-square test

^§^A standardized mean difference of ≤0.1 indicates a negligible difference between the two cohorts.

**Fig 1:**
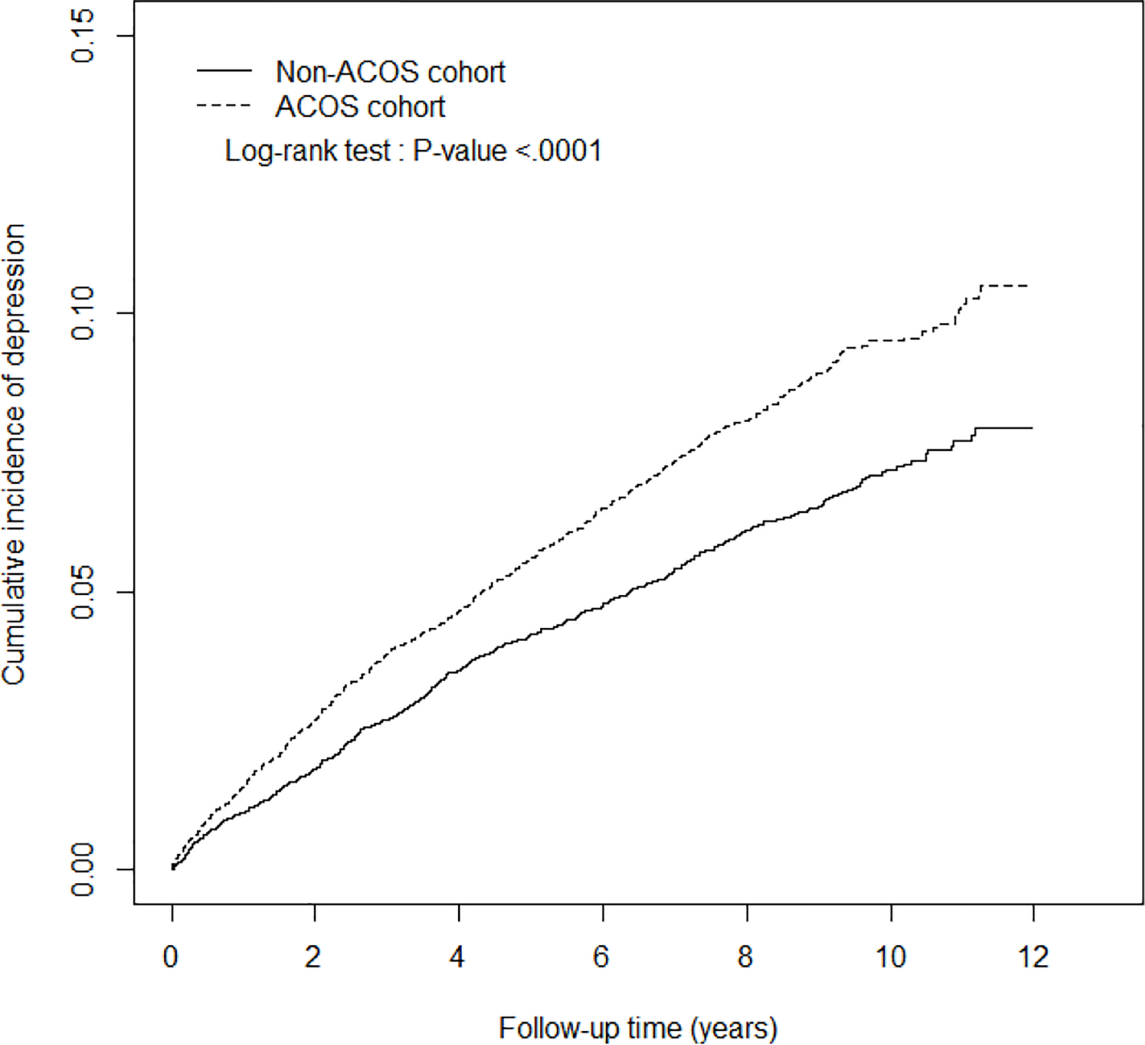
Cumulative incidence of depression in the ACOS (dashed line) and non-ACOS cohorts (solid line). ACOS, asthma–COPD overlap syndrome; COPD, chronic obstructive pulmonary disease.

The incidence of depression was 10.7 and 7.87 per 1,000 person-years in the ACOS and non-ACOS cohorts, respectively (Table 2). As shown in Fig 1, the cumulative incidence of depression was significantly higher in the ACOS cohort than in the non-ACOS cohort. After adjustment for age, sex, comorbidities, and ICSs and OSs use, the adjusted risk of depression was nearly 1.67-fold higher in the ACOS cohort than in the non-ACOS cohort (adjusted HR [aHR] = 1.67, 95% CI = 1.48–1.88). Stratified analysis indicated that compared with the non-ACOS cohort, the risk of depression in the ACOS cohort was statistically significant across all age groups, both sexes, and the comorbidity status. The adjusted risk of depression was 1.73-fold higher in female patients in the ACOS cohort (aHR = 1.73, 95% CI = 1.47–2.05) and 1.62-fold higher in male patients in the ACOS cohort (aHR = 1.62, 95% CI = 1.36–1.92) than in their corresponding counterparts in the non-ACOS cohort. Compared with the non-ACOS cohort, the aHRs for the risk of depression were 2.44 (95% CI = 1.45–4.11), 1.71 (95% CI = 1.39–2.10), and 1.61 (95% CI = 1.38–1.87) in patients aged 20–39, 40–64, and ≥65 years in the ACOS cohort. Among the study subjects without any comorbidity, ACOS patients exhibited a significantly higher adjusted risk of depression than subjects without ACOS (aHR = 2.36, 95% CI = 1.58–3.52). Among all subjects with at least one comorbidity, the ACOS patients exhibited a significantly higher adjusted risk of depression than the subjects without ACOS (aHR = 1.61, 95% CI = 1.42–1.82).

**Table 2:** Risk of depression between the ACOS and non-ACOS cohorts stratified by sex, age, and comorbidity (no/yes).

	ACOS							
		No			Yes		Compared to non-ACOS cohort	
Variables	Events	PY	Rate	Events	PY	Rate	Crude HR (95% CI)	Adjusted HR (95% CI)^†^
**Overall**	555	70477	7.87	733	68530	10.7	1.36(1.22, 1.51)***	1.67(1.48, 1.88)***
**Sex**								
Women	275	30236	9.10	389	30098	12.9	1.42(1.22, 1.66)***	1.73(1.47, 2.05)***
Men	280	40241	6.96	344	38433	8.95	1.28(1.10, 1.50)**	1.62(1.36, 1.92)***
**Age, year**								
20–39	24	4648	5.16	58	5281	11.0	2.12(1.32, 3.41)**	2.44(1.45, 4.11)***
40–64	178	28008	6.36	275	28733	9.57	1.50(1.25, 1.82)***	1.71(1.39, 2.10)***
≥65	353	37821	9.33	400	34516	11.6	1.24(1.07, 1.43)**	1.61(1.38, 1.87)***
**Comorbidity**^‡^								
No	46	10447	4.40	82	10185	8.05	1.82(1.27, 2.61)**	2.36(1.58, 3.52)***
Yes	509	60030	8.48	651	58345	11.2	1.31(1.17, 1.48)***	1.61(1.42, 1.82)***

ACOS, asthma–COPD overlap syndrome; COPD, chronic obstructive pulmonary disease; PY, person-year; Rate, incidence rate (per 1000 person-years); adjusted HR^†^: multiple Cox models included age, sex, each comorbidity, inhaled corticosteroids (ICSs), and oral steroids (OSs).

Comorbidity^‡^: Patients with any one of the comorbidities hypertension, diabetes, hyperlipidemia, IHD, sleep disorder, anxiety, alcohol-related illness, vitamin D deficiency, cancer, tubeculosis, rheumatoid arthritis, gout, biral hepatitis, and herpes simplex were classified as the comorbidity group

** p < 0.01

***p < 0.001

Table 3 presents the risk of depression associated with ICSs and OSs on in the ACOS cohort. Compared with the patients in the non-ACOS cohort, the ACOS patients without ICSs use had a 1.70-fold higher adjusted risk of depression (aHR = 1.70, 95% CI = 1.51–1.93). However, the adjusted risk of depression was non significant among the ACOS patients with ICSs use (aHR = 1.16, 95% CI = 0.95–1.41). Similarly, comparision of the subjects without ACOS; the aHRs for the risk of depression were 1.84 (95% CI = 1.55–2.19) and 1.12 (95% CI = 0.96–1.29) in the ACOS patients without and with OSs use, respectively.

**Table 3:** Adjusted hazard ratio for depression in the ACOS patients with and without ICSs and OSs use during the follow-up period.

Variables	N	Event	Rate	Crude HR (95% CI)	Adjusted HR (95% CI)
**Non-ACOS cohort**					Adjusted HR^a^
Without ICSs	10452	534	7.92	1.00	1.00
With ICSs	459	21	6.95	0.88(0.57, 1.36)	1.01(0.65, 1.57)
**ACOS cohort**					
Without ICSs	8075	594	12.2	1.53(1.36, 1.72)***	1.70(1.51, 1.93)***
With ICSs	2836	139	7.07	0.90(0.75, 1.09)	1.16(0.95, 1.41)
**Non-ACOS cohort**					Adjusted HR^b^
Without OSs	5617	319	8.75	1.00	1.00
With OSs	5294	236	6.94	0.79(0.67, 0.94)**	0.73(0.61, 0.87)***
**ACOS cohort**					
Without OSs	2426	223	15.7	1.78(1.50, 2.11)***	1.84(1.55, 2.19)***
With OSs	8485	510	9.38	1.07(0.93, 1.23)	1.12(0.96, 1.29)

ACOS, asthma–COPD overlap syndrome; COPD, chronic obstructive pulmonary disease; Rate, incidence rate (per 1,000 person-years); inhaled corticosteroids (ICSs), and oral steroids (OSs)

Adjusted HR^a^: multiple Cox models included age, sex, each comorbidity, and OSs

Adjusted HR^b^: multiple Cox models included age, sex, each comorbidity, and ICSs

** p < 0.01

***p < 0.001

The results of the univariable and multivariable Cox proportional hazards regression models for analyzing the risk of variables contributing to depression are shown in Table 4. The aHR of depression was increased 1.18-fold for men relative to women (95% CI = 1.06–1.33). The risk of depression was greater in patients with comorbidities, namely IHD (aHR = 1.22, 95% CI = 1.07–1.38), sleep disorder (aHR = 2.05, 95% CI = 1.82–2.30), anxiety (aHR = 1.23, 95% CI = 1.05–1.44), and alcohol-related illness (aHR = 1.55, 95% CI = 1.12–2.14). The risk of depression was less in patients with comorbidities, namely cancer (aHR = 0.62, 95% CI = 0.49–0.77), gout (aHR = 0.78, 95% CI = 0.68–0.89), viral hepatitis (aHR = 0.80, 95% CI = 0.65–0.99), herpes simplex (aHR = 0.68, 95% CI = 0.48–0.95) and [ICSs; OSs] (aHR = 0.72, 95% CI = 0.60–0.85; aHR = 0.66, 95% CI = 0.59–0.75). The risk of depression was not significant difference in patients having comorbidities associated with chronic diseases, namely hypertension (aHR = 1.11, 95% CI = 0.97–1.27), diabetes (aHR = 1.11, 95% CI = 0.98–1.27), hyperlipedemia (aHR = 1.04, 95% CI = 0.82–1.18), tuberculosis (aHR = 0.93, 95% CI = 0.75–1.16) and rheumatoid arthritis (aHR = 0.91, 95% CI = 0.41–2.03).

**Table 4:** HR of depression in Association with Sex, Age, and Comorbidities in Univariable and Multivariable Cox Regression Models.

	Crude		Adjusted^†^	
Variable	HR	(95% CI)	HR	(95% CI)
**ACOS**	1.36	(1.22, 1.51)***	1.67	(1.48, 1.88)***
**Sex (men vs women)**	1.40	(1.25, 1.56)***	1.18	(1.06, 1.33)**
**Age, years**	1.01	(1.00, 1.01)*	1.00	(0.99, 1.01)
**Baseline comorbidities (no vs yes)**				
Hypertension	1.28	(1.14, 1.43)***	1.11	(0.97, 1.27)
Diabetes	1.26	(1.12, 1.42)***	1.11	(0.98, 1.27)
Hyperlipidemia	1.23	(1.10, 1.37)***	1.04	(0.92, 1.18)
IHD	1.37	(1.23, 1.53)***	1.22	(1.07, 1.38)**
Sleep disorder	2.21	(1.98, 2.47)***	2.05	(1.82, 2.30)***
Anxiety	1.69	(1.45, 1.97)***	1.23	(1.05, 1.44)*
Alcohol-related illness	1.45	(1.05, 2.01)*	1.55	(1.12, 2.14)**
Vitamin D deficiency	-	-	-	-
Cancer	0.57	(0.45, 0.71)***	0.62	(0.49,0 .77)***
Tubeculosis	0.77	(0.62, 0.95)*	0.93	(0.75, 1.16)
Rheumatoid arthritis	0.92	(0.41, 2.05)	0.91	(0.41, 2.03)
Gout	0.79	(0.70, 0.90)***	0.78	(0.68, 0.89)***
Viral hepatitis	0.81	(0.66, 1.00)	0.80	(0.65, 0.99)*
Herpes simplex	0.69	(0.49, 0.96)*	0.68	(0.48, 0.95)*
Medicine				
Inhaled corticosteroids (ICSs)	0.74	(0.62, 0.87)***	0.72	(0.60, 0.85)***
Oral steroids (OSs)	0.79	(0.71,0 .88)***	0.66	(0.59, 0.75)***

Crude HR, relative hazard ratio; Adjusted HR^†^: multiple Cox models included age, sex, each comorbidity, inhaled corticosteroids (ICSs), and oral steroids (OSs).

*p<0.05

**p<0.01

***p<0.001

## Discussion

The ACOS–depression link is likely attributable to the deteriorating quality of life caused by lung disease. High scores for the major components of the COPD Assessment Test, such as dyspnea [23] and poor sleep with depression, are consistent with lower health-related quality of life (HRQoL). This finding is also applicable to ACOS patients [24]. Lower HRQoL [24, 25], higher admission frequency [25], and acute respiratory event occurrence [26] contribute to the higher risk of incident depression in ACOS patients, regardless of sex, age, and comorbidities—even in those without co morbidities or in young adults in our study. Previous studies have shown that ACOS patients with depressive symptoms [20] may be more prone to exacerbations [27] which subsequently lead to more depression [28], which is consistent with our study findings. Meanwhile, the comorbidities with chronic diseases (e.g., hypertension, diabetes, hyperlipidemia, tuberculosis, rheumatoid arthritis) were not associated with the higher risk of the depression. This finding implies that ACOS cohort primarily itself was associated with the event of the depression even without comorbidities. However, the events of the depression were not significant difference in patients receiving the ICSs/OSs between the ACOS and the non-ACOS cohorts.

In a multicenter, observational, cross-sectional study, Barrecheguren et al [29] reported that patients with COPD and a history of asthma before the age of 40 years were more likely to receive a diagnosis of ACOS patients compared with the pure COPD patients (non-ACOS). The incidence of depression was higher in the ACOS patients. Kumbhare et al [28] reported the characteristics and prevalence of ACOS in the United States. Depression in their ACOS group (45.9%) was higher than that in their control group (15.9%) and COPD (32.5%) and asthma (29.3%) groups. In the present study, after adjustment for age, comorbidities, and OSs/ICSs use, the risk of depression remained higher in the ACOS cohort than in the non-ACOS cohort agree with theses previous reports.

The non-ACOS cohort (e.g., pure COPD cohort) may have the less OSs/ICSs use, if proper treatment is given. The severe form of ACOS received the more OSs/ICSs. The OSs/ICSs [30, 31] may attenuate airway inflammation in ACOS cohort with exacerbation [32], thus leading to higher HRQoL which may counterbalance the event of the depression [13]. In our study, the non-ACOS cohort without the OSs/ICSs as reference, the risk of the depression in the patients receiving ICSs/OSs without significant differences between the ACOS cohort and the non-ACOS cohort (Table 3) support this speculation. In recent Spain study [14], the defined the ACOS cohort based on the patients who had received a physician confirmed diagnosis of both asthma ([ICD-9] code: 493) and COPD (ICD-9 codes:491, 492, and/or 496), and combined with medication (ATC [Anatomical Therapeutic Chemical] code: R03, Antiasthmatics [33]). In their study, the frequency of the depression between the ACOS cohort (n = 5,093) and COPD cohort (n = 22,778) was without significant difference. The result of this previous study support our finding. In an European report, the frequency of the anxity/depression in the patients receiving the ICSs/OSs with the ACOS cohort (n = 703; 34.3%) is nearly the same as in the COPD cohort (n = 519,35.8%) [34] in line with these findings.

Our ACOS cohort with comorbidities (e.g., anxity, sleep, alcohol-related diseases, ischemic heart disease) exhibited a higher risk of incident depression, which is consistent with previous findings [9]. In versa, the comorbidities such as cancer, gout, viral hepatitis and herpes simplex were associated with the less risk of depression. A possible explanation is that these patients received the OSs for the acute gout attack [35] and ICSs for the ACOS with excerbation [36], the additive effect of the OSs on the ICSs [37] may attenuate the frequency of the admission to the acute respiratory ward [38]. Thus, the events of the depression in the ACOS cohort with comorbidities (e.g., cancer, gout) were less comparison of the non-ACOS cohort.

This observation is likely to reflect the complex effects of ICSs/OSs [12, 35] on the central nervous system and the probable interplay among individual susceptibility [39], disease factors (e.g., comorbidities), and external environmental stressors (e.g., alcohol) [33]. Therefore, in different study cohorts, steroid use at different doses or for different durations has different impacts on the risk of depression.

Patients in the COPD-Depression cohort were more likely to have a COPD-related hospitalization and an emergency room (ER) visit compared to the COPD-Only cohort [40]. Compared with the formular without ICSs, the formular with ICSs was associated with more favorable COPD-related outcomes and lower COPD-related utilization and medical costs among patients with COPD and comorbid depression. [41] Similarly, the higher frequency of admission to ER [21], intensive care unit in the ACOS cohort [20]. Therefore, the higher risk of the depression in the ACOS cohort was in the acute respiratory ward [38] and related to a worse course [20,42]. Moreover, comorbid depression of patients with ACOS cohort is 1.67 fold higher than the non-ACOS cohort (e.g., pure COPD cohort) in our study. Despite its negative impact, depression often remains unrecognized and untreated in ACOS patients. This study alert the physician to early detect the ACOS [29] among the patients with the COPD [14, 21] or asthma [29, 43].

In this study, the incidence of depression revealed significant increase in the ACOS cohort without ICSs/OSs. By contrast, the risk of the depression was without significant difference in the ACOS cohort with ICSs/OSs use [41]. Thus, for the daily care, we may try to treat the ACOS cohort using the adequate dose of the ICSs/OSs in the early course for avoiding the incident of the depression. Therefore, this policy may have the benefit for the prevention of the ACOS-depression in the late course of the ACOS cohort with lower HRQoL [25, 37, 42, 44]; however, this speculation warrant randomized controlled trial.

### Strengths

Depression was diagnosed using the Automated Geriatric Examination for [44] Computerised Assisted Taxonomy. Data on life events were collected using the Taiwanese version of the Life Events and Difficulties Schedule [45, 46].

The healthcare system, such as the Taiwan Association Against Depression, has established a community-based walk-in screening service for depression. This policy may avoid diagnosis bias or the underdiagnosis of depression. A multidisciplinary system of diagnosis and care for asthma, COPD, and ACOS has also been established in Taiwan [18]. Furthermore, in response to the increasing demand, in 2007, the Executive Yuan launched the National 10-year Long-Term Care Plan in Taiwan. This plan guarantees that disabled seniors and other physically and mentally handicapped people receive appropriate services to boost their independent and autonomous life skills, upholding human dignity and increasing quality of life. The available services include home nursing, rehabilitation at home and in the community, respite services, care services, assistive device purchase or rental assistance, aid with home improvements to make one’s environment barrier-free, and long-term care institutions. This multidisciplinary team may avoid the bias of the initial diagnosis or follow up of depression [45, 46].

### Limitation

Ethnicity, marital status, religion, education, living conditions, offspring, physical illness, and other drugs (e.g., Inderal) that may induce depression were not included in the analysis. The diagnosis of ACOS is challenging for physicians. These confounding factors are the limitations of this study.

### Conclusion

Regardless of sex, age, or comorbidities, the risk of incident depression is higher in ACOS patients, even in those without comobordities or young adults. The events of the depression were not significant difference in patients receiving the ICSs/OSs between the ACOS and non-ACOS cohorts.

## Supporting information

S1 STROBE Checklist Checklist of items that should be included in reports of observational studies. (DOC)

## Abbreviations

COPD: chronic obstructive pulmonary disease
ACOS: Asthma–chronic obstructive pulmonary disease syndrome
OS: oral steroid
ICS: inhaled corticosteroid
NHIRD: National Health Insurance Research Database
LHID: Longitudinal Health Insurance Database
ICD-9-CM: International Classification of Diseases, Ninth Revision, Clinical Modification
HR: hazard ratio
CI: confidence interval
